# Angiotensin-converting enzyme gene insertion/deletion polymorphism and risk of ischemic stroke complication among patients with hypertension in the Ethiopian population

**DOI:** 10.3389/fneur.2023.1093993

**Published:** 2023-03-22

**Authors:** Addisu Melake, Nega Berhane

**Affiliations:** ^1^Department of Biomedical Science, College of Health Science, Debre Tabor University, Debre Tabor, Ethiopia; ^2^Department of Medical Biotechnology, Institute of Biotechnology, University of Gondar, Gondar, Ethiopia

**Keywords:** angiotensin-converting enzyme, genotype, hypertension, ischemic stroke, polymerase chain reaction

## Abstract

**Background:**

Ischemic stroke is a complicated, multifaceted condition brought on by a confluence of vascular, environmental, and genetic variables. The burden of ischemic stroke is currently rising in terms of death, morbidity, and disability worldwide. Genetic variables also play significant roles in the pathophysiology of hypertension and ischemic stroke in addition to the greatest effects of demographic, clinical, and behavioral risk factors. The key functional variation of the ACE gene that has drawn the most interest is the ACE I/D variant. Even though the ACE gene I/D polymorphism has been widely studied, the findings of investigations on the involvement of this polymorphism in ischemic stroke were contradictory and provide conflicting data. The goal of this study was to look into the effect of the ACE gene I/D polymorphism on the risk of ischemic stroke in patients with hypertension.

**Methods:**

A hospital-based case–control study was carried out in 36 cases of patients with hypertensive IS and 36 age- and sex-matched healthy controls. Clinical and biochemical parameters were measured to assess the associated risk factors. The DNA was isolated from blood samples, and the ACE I/D genotypes were identified using polymerase chain reaction and analyzed by agarose gel electrophoresis.

**Results:**

The ACE-DD genotype (OR = 3.71, 95% CI = 1.02–13.5; *P* < 0.05) and D allele (OR = 2.07, 95% CI = 1.06–4.03; P < 0.05) were significantly more common in patients than in controls, indicating that it is a risk factor for the development of ischemic stroke in hypertensive individuals.

**Conclusion:**

There is a significant correlation between the ACE gene I/D polymorphism and the development of ischemic stroke in patients with a history of hypertension in the Ethiopian population.

## 1. Introduction

Ischemic stroke (IS) is the loss of brain tissue caused by a cessation of blood supply to a region of the brain brought on by an obstruction of a carotid or vertebral artery, or distal branches of the anterior, middle, or posterior cerebral arteries (1). It is the primary cause of adult disability and the second-leading cause of mortality in the world, accounting for ~ 10% of all deaths with 5.5 million people dying annually (2). According to data from the 2010 Global Burden of Diseases, Injuries, and Risk Factors Study (GBD), stroke is the most common cardiovascular disease (CVD) that results in death and disability in sub-Saharan Africa (SSA) and other low- and middle-income countries (LMICs) (3). Risk factors of IS may be divided into two categories: modifiable and non-modifiable. Age, sex, family history, and race/ethnicity are risk factors that cannot be modified, but hypertension, smoking, diet, and physical inactivity are some of the risk factors that can be modified (4). Due to health changes associated with constantly evolving social, economic, and demographic trends, the SSA and LMICs may be more impacted by the high burden of IS and other vascular illnesses. The population's shifting exposure to risk factors and their inability to pay the high cost of IS treatment are two additional reasons why the poor are becoming more and more impacted by this illness (5).

The 2017 WHO data reported that 6.23% of all fatalities in Ethiopia were due to stroke. In addition, the nation's age-adjusted stroke death rate is 89.82 per 100,000 of the population. The stroke burden will increase in the upcoming years as a result of poor healthcare-seeking behavior and insufficient neurologic therapies, according to previous data on the stroke trend (6). According to hospital-based research, stroke accounts for 24% of all neurologic hospitalizations in Ethiopia, making it one of the major causes of morbidity and death. Furthermore, due to changes in lifestyle and demographics that have an impact on the population's epigenetic makeup, the incidence of risk factors for stroke has been rising in the Ethiopian population (7).

Hypertension is the most significant modifiable risk factor for IS, increasing the relative risk by 3.1 times for men and 2.9 times for women, where the incidence of stroke rises proportionately with both systolic and diastolic blood pressure (8). Since HTN and IS share fundamental physiological regulatory systems, the causes of elevated blood pressure that operate through RAAS may be linked to IS. In addition, the atherosclerotic process involves RAAS in vascular remodeling, the production of oxidative stress, and inflammation that have shown a potential relationship with the condition (9). A number of RAAS gene polymorphisms, including those in the aldosterone synthase (CYP11B2), angiotensinogen (AGT), angiotensin II type 1 receptor (AT1R), and angiotensin-converting enzyme (ACE) genes, have been found to be strongly linked to hypertension and ischemic stroke. The most well-known and extensively researched variants of these polymorphisms are the ACE I/D polymorphisms (10).

The ACE is a membrane-bound dipeptidyl carboxypeptidase ectoenzyme found in the endothelium lining of blood arteries throughout the body, where it plays a crucial role in the proliferation of vascular smooth muscle cells by converting angiotensin I to angiotensin II (11). The human ACE gene, which spans 21 kb and has 26 exons and 25 introns, is located on the long arm (q) of chromosome 17 (17q23.3). The ACE gene's I/D polymorphism results from the insertion (I) or deletion (D) of a 287-base pair (bp) Alu sequence in intron 16, resulting in three genotypes: II homozygote, ID heterozygote, and DD homozygote (12). The ACE insertion/deletion (I/D) gene polymorphism (rs4646994) was shown to have a high association with the level of plasma ACE since it accounted for 47% of the overall phenotypic variance of ACE activity. According to some studies, those with the II genotype had lower ACE concentrations than people with the DD genotype (13). The DD genotype was linked to elevated ACE levels and activity, which consequently caused a spike in blood pressure by increasing the production of angiotensin II, starting the constriction of blood vessels, and also increasing the reabsorption of water and sodium by the kidneys, along with elevating blood volume and blood pressure that causes HTN-induced IS (14).

Numerous case–control studies on ACE I/D polymorphism and the risk of IS in various ethnic populations had been conducted. This led to the hypothesis that ACE I/D may be a candidate gene and that the DD genotype is correlated with IS (10). However, the findings of investigations on the involvement of this polymorphism in IS are contradictory and provide conflicting data as certain studies established a link while others did not (15). Furthermore, though a number of studies were conducted to estimate the prevalence, risk factors, and outcome of IS in the Ethiopian population (16), there are no reported data about the effect of ACE gene polymorphism on the occurrence and progression of the disease. Keeping all the aforementioned factors in mind, the purpose of this study was to determine the relationship between ACE gene I/D polymorphism and the risk of ischemic stroke complications among patients with hypertension in the Ethiopian population.

## 2. Methods

### 2.1. Study participants

**Figure 1:**
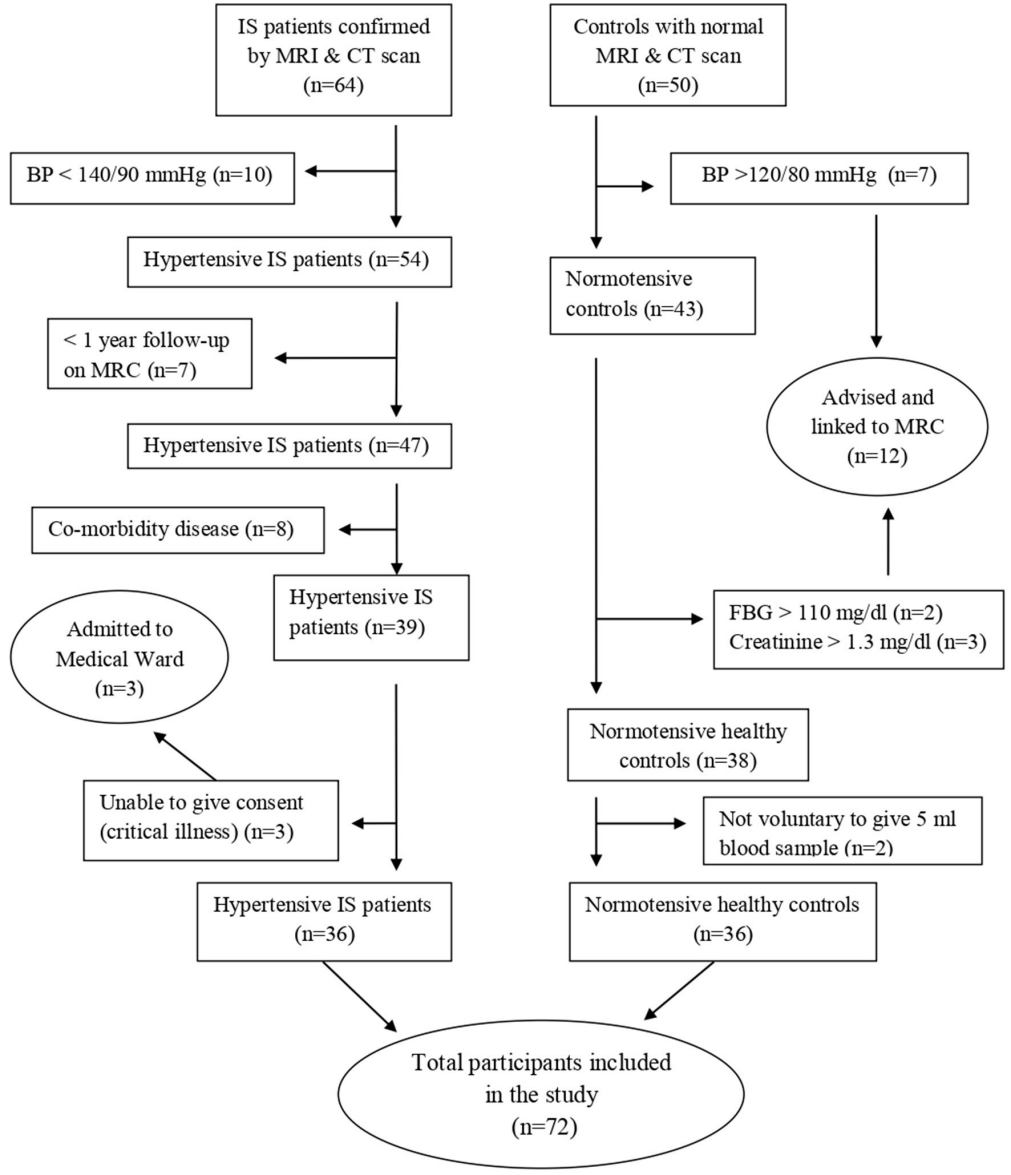
Flow diagram of the study participant selection process.

A hospital-based matched case–control study was conducted from May to August 2022 in Debre Tabor Referral Hospital. It has a follow-up medical referral clinic (MRC) for major chronic illnesses, including IS and HTN, in which treatment and follow-up for those patients take place. All patients who visit MRC were the source population, and patients who are under follow-up for HTN with IS complications were study subjects. The study included a total of 72 participants of both sexes, consisting of 36 patients with hypertensive IS and 36 normotensive healthy control groups (Figure 1).

### 2.2. Inclusion and exclusion criteria

Patients with IS secondary to HTN, who had been confirmed by computed tomography (CT) scans and magnetic resonance imaging (MRI), were recruited into this study. The study included patients who had been receiving follow-up care at MRC for at least 1 year. Controls were age- and sex-matched normotensive volunteers who were available during the study period. They were healthy individuals with normal brain imaging from the same geographical location and social status (Figure 1).

Patients who are diagnosed with hemorrhagic stroke, transient ischemic attack, hepatic and renal disease, cardiac source of embolization, secondary HTN, or chronic bacterial or viral infection were excluded. Patients who are unable to respond or are not willing to sign informed consent were also excluded from this study.

### 2.3. Data collection methods

The socio-demographic characteristics of both patients and healthy control subjects were obtained through a semi-structured questionnaire. Portable digital scales and portable stadiometers were used to determine body weight and height, respectively. Body mass index (BMI) is computed by dividing weight (in kilograms) by height (in meters square). Participants were classified as underweight (BMI < 18.5 kg/m^2^), healthy (18.5–25 kg/m^2^), overweight (25.0–29.9 kg/m^2^), or obese (≥30 kg/m^2^) based on their BMI (17). A digital instrument was used to measure blood pressure in the sitting stance after 5 min of rest, and the mean of three readings was used to compute SBP and DBP. Participants were categorized as hypertensive if their SBP was 140–159 mmHg and/or DBP was 90–99 mmHg (grade 1) or SBP was ≥ 160 mmHg and/or DBP was ≥ 100 mmHg (grade 2) or if they used antihypertensive medication; as having high normal BP, if SBP was 130–139 mmHg and/or DBP was 85–89 mmHg; and as having normal BP, if SBP was < 130 mmHg and DBP was < 85 mmHg (18).

### 2.4. Sample collection and laboratory methods

All participants, including patients and healthy controls, had a blood sample of 5 ml taken from the median cubital vein by laboratory staff under safety procedures. From the 5 ml sample, 3 ml was retained in the test tube without anticoagulants to allow the blood to clot. The tubes were then centrifuged to extract the serum, which was then collected into new tubes for biochemical tests. Enzymatic analyses of TC, TG, LDL, HDL, creatinine, and glucose were performed on each test in the Debre Tabor Referral Hospital diagnostic laboratory using the Dimension EXL 200 fully automated analyzer. The results were then scored by an investigator blinded to the sample withdrawal condition and experimental groups. If the fasting plasma glucose concentration is >126 mg/dl, then diabetes mellitus has been identified (19). Dyslipidemia can be defined if TC, TG, and LDL levels are above 200 mg/dl, 150 mg/dl, and 100 mg/dl, respectively, and the HDL level is below 60 mg/dl (20). Kidney disease is diagnosed if the blood creatinine concentration is >1.3 mg/dl for men and >1.1 mg/dl for women (21).

**Figure 2:**
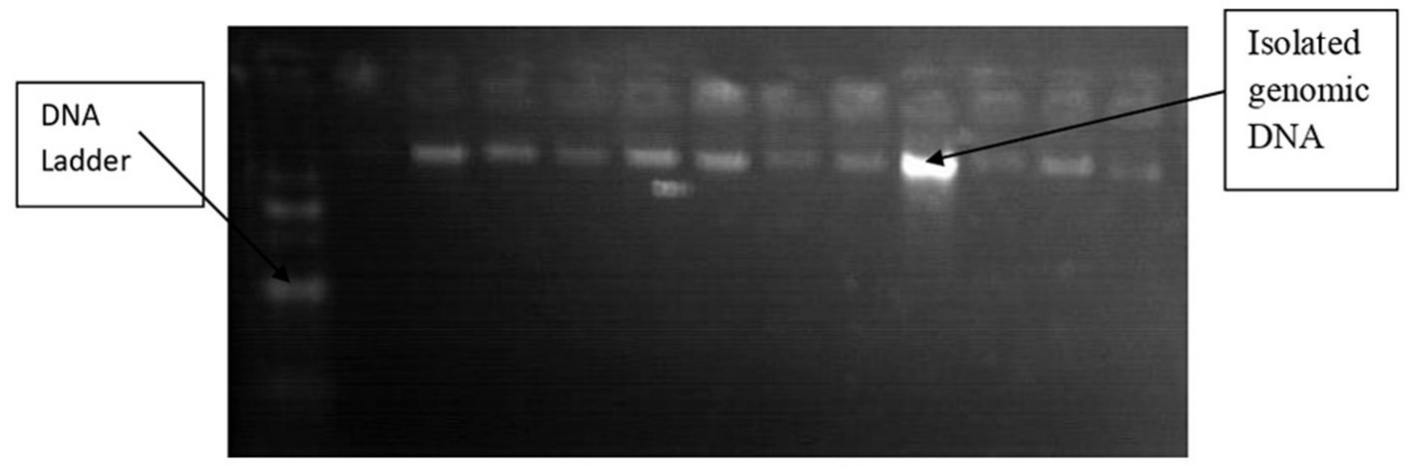
Agarose gel (1%) electrophoresis showing the quality of isolated genomic DNA.

In the molecular biology laboratory at the University of Gondar, genomic DNA was extracted from the remaining 2 ml of samples collected in EDTA-containing tubes from each participant. The non-enzymatic salting-out approach (22) was used to isolate DNA from EDTA-anticoagulated blood from both patients and controls. The blood was then put into a clean 1.5 ml Eppendorf tube. By lysing and eliminating them with a buffer solution, red blood cells were removed. To lyse white blood cells, a nuclear lysis buffer solution was used. Thereafter, to precipitate and remove proteins, 6 M NaCl of a highly concentrated salt was applied. After freezing with isopropanol and washing with 70% ice-cold ethanol, the DNA was precipitated. Thereafter, Tris-EDTA (TE) buffer was used to dissolve genomic DNA. The quality of isolated genomic DNA was verified utilizing 1% agarose gel electrophoresis (Figure 2), and the sample was kept at −20 °C until it was needed (23).

Using specific primers (5′- CTG GAG ACC ACT CCC ATC CTT TCT-3′ and 5′- GAT GTG GCC ATC ACA TTC GTC AGA T-3′, respectively), direct PCR was used to identify the I/D alleles of the ACE gene polymorphism (24). A final volume (25 μl) of the PCR reaction mixture was prepared by combining 12.5 μl of master mix (MgCl2, dNTPs, PCR buffer, and Taq polymerase), 1 μl of forward primer, 1 μl of reverse primer, 2 μl of each sample, and 8.5 μl of PCR-grade water. The first denaturation step of the PCR amplification was set at 95°C for 5 min. The DNA was then amplified for 35 cycles with denaturation at 94°C for 30 s, annealing at 58°C for 30 s, extension at 72°C for 1 min, and a final extension at 72°C for 5 min (25).

**Figure 3:**
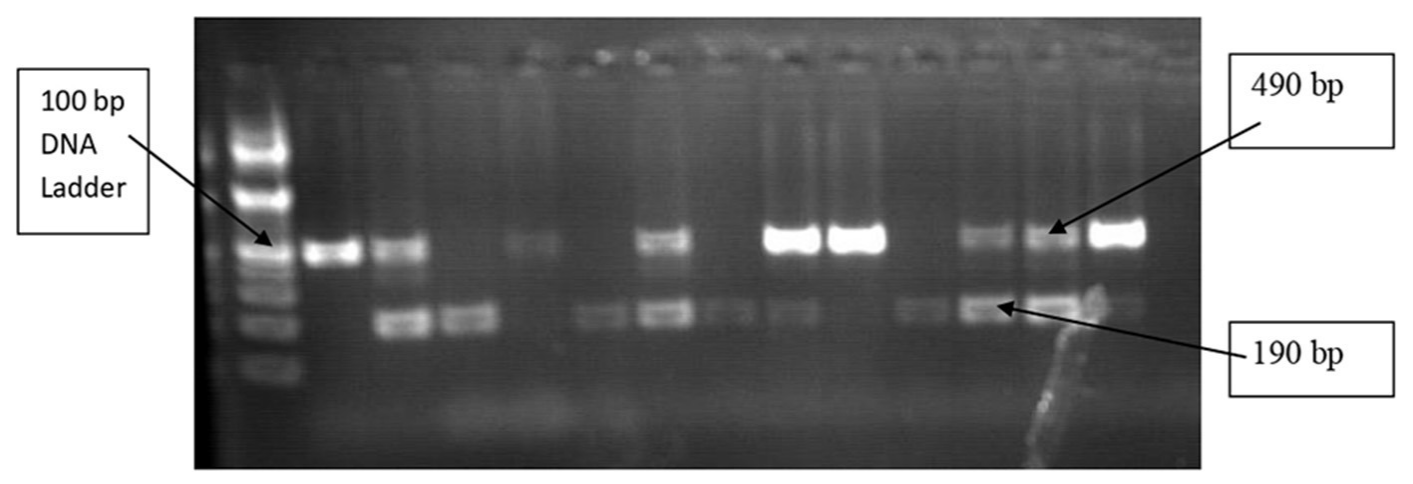
Agarose gel (2%) electrophoresis showing PCR products of the ACE I/D gene.

ACE I/D genotypes of 490 bp band (II), 190 bp band (DD), and both 490 bp and 190 bp band (ID) PCR products were electrophoretically separated for 50 min at 120 V on a 2% agarose gel (Figure 3). The PCR-amplified products (12 μl) were mixed with 3 μl of loading dye before being injected into the agarose gel wells. DNA ladders, which are molecular weight markers, were electrophoresed along with the DNA fragments to estimate the sizes of fragments of interest, and 3 μl of 2% ethidium bromide was also used for staining. In 1X tris acetate EDTA (TAE) buffer, electrophoresis was performed, and a UV transilluminator was used to visualize the gel (26).

### 2.5. Statistical analysis

The data were analyzed using STATA version 14. The means and standard deviations (x + s) were used to show quantitative data. A *t*-test for independent samples was applied to compare continuous variables between patients with hypertensive IS and healthy controls. The chi-square test was used to compare the distribution of genotype and allele frequencies. The risk correlations of ACE gene I/D polymorphisms with hypertensive IS were evaluated using logistic regression with a 95% confidence interval (CI). A one-way ANOVA was used to compare the relationships between ACE genotypes and clinical factors. Statistical significance was defined as a *p*-value of < 0.05.

## 3. Results

### 3.1. Socio-demographic and clinical characteristics

The distribution by sex and age was similar between hypertensive IS cases and normotensive control groups. Of the total 36 participants with hypertensive IS, 19 (52.8%) were male patients and 17 (47.2%) were female patients. Similarly, among the 36 healthy control groups, 18 (50 %) were male patients and 18 (50%) were female patients. The mean ages of the study groups were 59.4 ± 12.1 and 58.2 ± 6.9 for cases and controls, respectively. The clinical risk factors of IS such as systolic blood pressure (SBP), diastolic blood pressure (DBP), total cholesterol (TC), triglycerol (TG), LDL-cholesterol, and HDL-cholesterol levels were significantly higher in patients when compared to controls. However, there were no appreciable variations in blood creatinine levels, fasting blood glucose (FBG), or body mass index (BMI) between the two groups (Table 1).

**Table 1:** Demographic, clinical, and behavioral characteristics of the study participants in Debre Tabor Referral Hospital, Northwest Ethiopia, 2022.

**Variables**	**IS (*n =* 36)**	**Control (*n =* 36)**	***P*-value**
Sex (M/F)	19/17	18/18	0.8136
Age (yr)	59.4 ±12.1	58.2 ± 6.9	0.6177
Family history of HTN (%)	27.8 %	19.4 %	0.4051
Family history of IS (%)	16.7 %	8.3 %	0.2850
BMI (Kg/m^2^)	23.6 ± 4.7	23.1 ± 4.2	0.6471
SBP (mmHg)	147.4 ± 6.0	116.2 ± 3.9	< 0.001^*^
DBP (mmHg)	91.3 ± 3.8	76.0 ± 4.0	< 0.001^*^
FBS (mg/dl)	91.0 ± 18.9	89.9 ± 5.7	0.7503
Total Cholesterol (mg/dl)	190.5 ± 47.6	153.4 ± 46.2	< 0.001^*^
Triglyceride (mg/dl)	135.4 ± 37.3	105.5 ± 37.1	< 0.001^*^
LDL-Cholesterol (mg/dl)	94.7 ± 32.6	68.5 ± 28.2	< 0.001^*^
HDL-Cholesterol (mg/dl)	43.8 ± 8.2	55.5 ± 11.5	< 0.001^*^
Creatinine (mg/dl)	0.79 ± 0.13	0.75 ± 0.12	0.2214
Smoking habit (yes/no)	6/30	2/34	0.1336
Alcohol intake (yes/no)	20/16	17/19	0.4793
Salt intake (yes/no)	35/1	32/4	0.1613
Physical exercise (yes/no)	3/33	7/29	0.1728
Stress (yes/no)	24/12	19/17	0.2296

* A P-value of < 0.05 is considered statistically significant. BMI, body mass index; SBP, systolic blood pressure; DBP, diastolic blood pressure; FBG, fasting blood glucose; LDL, low-density lipoprotein; HDL, high-density lipoprotein; HTN, hypertension; IS, ischemic stroke.

### 3.2. Distribution of ACE genotypes and allele frequencies

**Figure 4:**
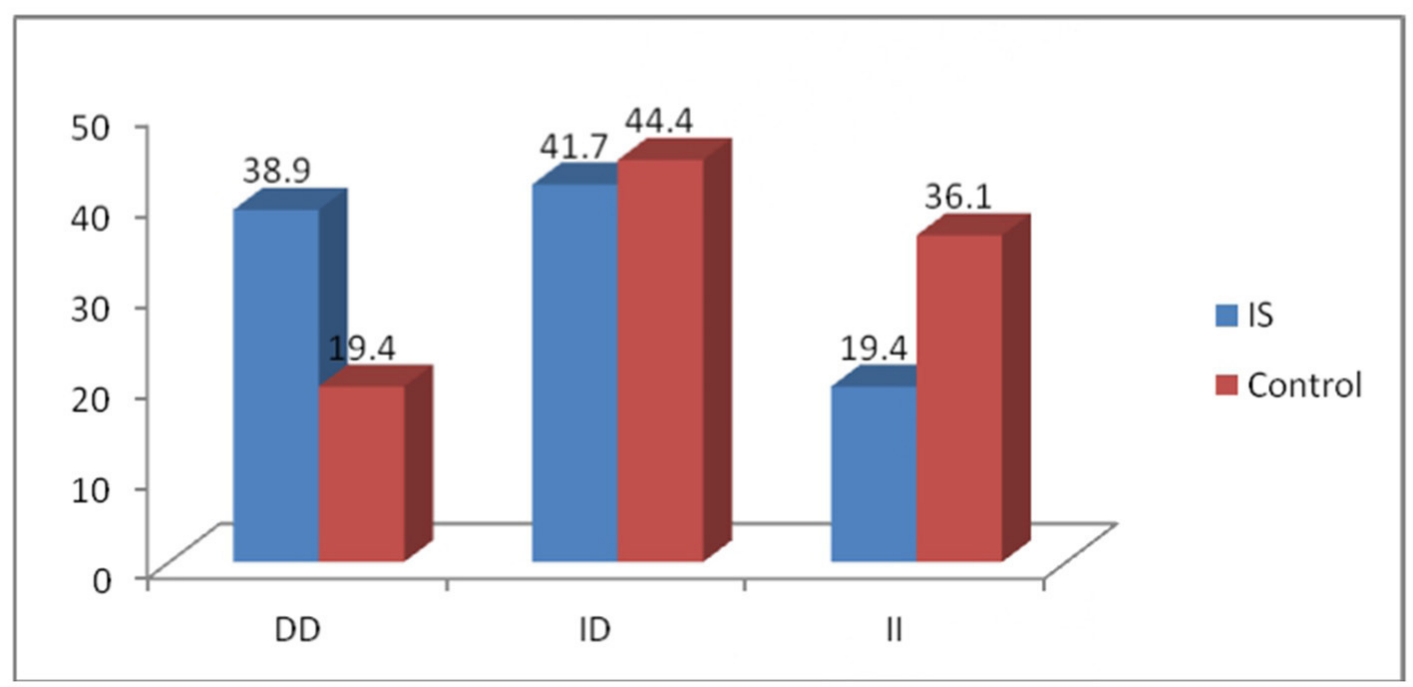
Distribution of the ACE I/D genotype in cases and controls.

The frequencies of the DD, ID, and II genotypes were 38.9, 41.7, and 19.4%, respectively, in the patient group, whereas they were 19.4, 44.4, and 36.1%, respectively, in the control group (Figure 4). Genotype distributions in the case and control groups were consistent with the Hardy–Weinberg equilibrium (*P* > 0.05). The D and I allele frequencies in patients were 0.60 and 0.40, respectively, while they were 0.42 and 0.58 in controls. The distribution of ACE genotype polymorphism between the two groups shows a significant difference (*P* < 0.05). Furthermore, compared to the control group, patients had a greater frequency of the homozygous DD genotype (odds ratio [OR] = 3.71; 95% confidence interval [CI] = 1.02–13.5; *P* < 0.05). The allelic frequencies showed high significance between the two groups, in which the D allele was two times higher than the I allele in patients (OR = 2.07; 95% CI = 1.06–4.03; *P* < 0.05) compared to controls. However, compared to healthy controls, ACE genotype II was less common in patients with hypertension (Table 2).

**Table 2:** Distribution of ACE genotypes and allele frequencies of the study participants in Debre Tabor Referral Hospital, Northwest Ethiopia, 2022.

**Genotype**	**IS (*n =* 36)**	**Control (*n =* 36)**	**OR (95% CL)**	***p*-value**
DD	14 (38.9 %)	7 (19.4 %)	3.71 (1.02–13.5)	0.046^*^
ID	15 (41.7 %)	16 (44.4 %)	1.74 (0.54–5.54)	0.348
II	7 (19.4 %)	13 (36.1 %)	Ref	
**Allele Frequency**				
D	43 (59.7 %)	30 (41.7 %)	2.07 (1.52–3.22)	0.031^*^
I	29 (40.3 %)	42 (58.3 %)	Ref	

* A P-value of < 0.05 is considered to be statistically significant. Ref, reference; CL, confidence level; OR, odds ratio.

### 3.3. Association between ACE genotypes and clinical parameters

Table 3 shows the association of the ACE I/D genotype with the clinical parameters of patients with hypertensive IS and normotensive healthy controls. The ACE genotypes (DD, ID, and II) in the study groups were assessed with FBG, blood pressure, and lipid profiles. All the aforementioned clinical variables were not found to be significant with the genotypes in the study groups (*P* > 0.05). However, the SBP and DBP are higher in the ACE-DD genotype than in the ID and II genotypes.

**Table 3:** Association of ACE I/D genotype with clinical characteristics in Debre Tabor Referral Hospital, Northwest Ethiopia, 2022.

	**Genotypes**			
**Variables**	**DD (*****n** =* **21)**	**ID (*****n** =* **31)**	**II (*****n** =* **20)**	***p*** **-value**
Sex (M/F)	57/44	36/39	30/30	0.6479
Age (yr)	58.2 ± 11.8	58.9 ± 9.7	59.1 ± 8.1	0.2947
BMI (Kg/m^2^)	23.7 ± 4.3	23.4 ± 4.1	22.8 ± 5.1	0.8263
SBP (mmHg)	135.6 ± 16.0	131.5 ± 17.8	128.2 ± 14.7	0.2167
DBP (mmHg)	85.4 ± 8.7	83.4 ± 9.4	82.2 ± 7.3	0.2280
FBS (mg/dl)	86.8 ± 9.8	90.9 ± 14.2	93.7 ± 16.6	0.5799
TC (mg/dl)	164.7 ± 41.1	179.8 ± 54.1	167.3 ± 53.5	0.7213
TG (mg/dl)	111.1 ± 41.1	128.4 ± 49.5	117.9 ± 30.2	0.6339
LDL-C (mg/dl)	90.8 ± 40.2	78.9 ± 32.7	76.2 ± 23.6	0.6194
HDL-C (mg/dl)	50.5 ± 11.3	48.2 ± 11.7	51.1 ± 11.7	0.8025
Creatinine (mg/dl)	0.79 ± 0.16	0.77 ± 0.12	0.75 ± 0.12	0.2451

^*^A P-value of < 0.05 is considered to be statistically significant.

## 4. Discussion

Ischemic stroke is a complex and heterogeneous disease with multiple etiologies and significant clinical manifestations (27). Even though conventional risk factors like diabetes, smoking, hypertension, and dyslipidemia were thought to be more important than inherited risk factors, recent case–control studies and meta-analyses have shown that genetic risk factors and genetic background have a significant impact on IS susceptibility (28). In the present study, the DD genotype and D allele had significantly higher frequencies in patients than in controls (Table 2, Figure 4). This finding is consistent with a meta-analysis conducted in a Caucasian population that included 22 case–control studies and discovered that patients with the DD genotype were more likely to have IS than those with the II genotypes (OR = 1.28, 95% CI = 1.05 to 1.55; *P* < 0.001) (29). Another meta-analysis with an encouraging result found that there was a substantial link between the D allele and IS in 105 relevant studies, encompassing 18,258 IS cases and 28,768 healthy controls (OR = 1.354; 95% CI = 1.272-1.440; *P* < 0.001). This meta-analysis suggests that although the statistical relevance for Caucasians is questionable, the ACE I/D polymorphism may be a genetic risk factor for IS, especially in Asians (30). Other research studies revealed that ACE-DD was connected to a high occurrence of IS in people from India (31), Iraq (32), and North India (33).

The exact mechanism by which the insertion/deletion mutation in the ACE gene increases the risk of HTN and IS was unknown (30). Extracellular volume and the homeostasis of the vascular wall are mediated by ACE, a crucial enzyme in the RAAS, which catalyzes the conversion of decapeptide angiotensin I into octapeptide angiotensin II. Numerous studies have shown that angiotensin II influences atherosclerotic changes and plaque rupture through a variety of mechanisms, including vasoconstriction and the expansion of vascular smooth muscle cells, which promote peripheral resistance of blood vessels (34). It has been shown that human RAAS activation worsens ischemia-induced brain injury mainly by stimulating atherosclerosis, reducing cerebral blood flow, and increasing oxidative stress, which results in a hypertension-induced IS complication (35). In addition, ACE-DD carriers have a higher level of angiotensin II than non-carriers, which affects the function of endothelial cells in a number of ways, including promoting endothelial cell apoptosis, raising vascular endothelial growth factor, and impairing the production of nitric oxide. This results in a higher risk of HTN and its associated IS complications (36).

Furthermore, the findings of this study disagree with the case–control study conducted in North Sumatra, Indonesia, which included a total of 78 patients with IS of both sexes, consisting of 43 patients with hypertension and 35 patients with normotension. Based on the allele and its genotype, there is no significant correlation between the ACE gene polymorphism and HTN in patients with IS. In this study, the I allele (72.1%) of the ACE gene polymorphism was more dominant than the D allele (27.9%) in patients with hypertensive IS (37). In addition, research conducted in South India (38) showed a link between the ACE II genotype and the I allele, which was exacerbated by factors such as smoking and diabetes among patients with IS. Other studies conducted in populations from Turkey (39) discovered no link between the ACE gene's I/D polymorphism and IS. The varying distribution frequencies of the ACE I/D polymorphism, which are impacted by regional and racial characteristics as well as ethnic variances, may be the reason for the controversial findings among the various ethnic communities (40). Different study methodologies, bias in selection, various matching criteria, or the kind of stroke might all be contributing variables (41).

In addition to the association between ACE polymorphism and IS, the contribution of other risk factors, such as the relation between ACE gene I/D polymorphism and patients with HTN without known histories of stroke, was studied in different populations (42). However, there are conflicting results regarding ACE polymorphisms and HTN risk (43). Studies conducted in populations from Pakistan (42) and Brazil (44) showed that ACE-DD was associated with a high incidence of HTN. Contradictory results were found from a study conducted in Afro-Brazilian and Caucasian populations (45) and the Peruvian elderly population (43), which showed no association between the ACE gene I/D polymorphism and HTN. Therefore, the controversial results about the association of ACE with hypertension and other cerebrovascular diseases in different populations may be due to interactions between genetic and some environmental factors that explain the complexity of genetic architecture (46). Inconsistencies were still visible between researchers about the ACE gene I/D polymorphism in hypertension and ischemic stroke complications. Therefore, further research is still required; for instance, a third group would be needed to conclusively show the relationship between patients with HTN without a known history of ischemic stroke, patients with hypertension with IS complications, and normotensive healthy controls.

## 5. Conclusion

The current study found that the ACE I/D gene of the DD genotype and D allele is associated with a high risk of IS complications in patients with hypertension. As a result, the ACE I/D gene polymorphism can be used as a biomarker for the early diagnosis and detection of IS. Further studies with a large sample size are required to comprehend the correlation between the ACE gene and IS.

## Data availability statement

The raw data supporting the conclusions of this article will be made available by the authors, without undue reservation.

## Ethics statement

The studies involving human participants were reviewed and approved by the University of Gondar Ethical Rievew Board. The patients/participants provided their written informed consent to participate in this study.

## Author contributions

AM prepared the final draft of the manuscript. NB critically reviewed the article and gave final approval for the version to be published. Both authors contributed to the article and approved the submitted version.

## References

[1] Wang J Sun Z Yang Y Wu J Quan W Chen X Ni P Li D. Association of laboratory parameters and genetic polymorphisms with ischemic stroke in Chinese Han population. Exp Ther Med. (2021) 21:1–9 10.3892/etm.2021.9921 33790999 PMC8005697

[2] Asres AK Cherie A Bedada T Gebrekidan H. Frequency, nursing managements and stroke patients' outcomes among patients admitted to Tikur Anbessa specialized hospital, Addis Ababa, Ethiopia a retrospective, institution based cross-sectional study. Int J Afr Nurs Sci. (2020) 13:100228. 10.1016/j.ijans.2020.100228

[3] Fekadu G Chelkeba L Kebede A. Risk factors, clinical presentations and predictors of stroke among adult patients admitted to stroke unit of Jimma university medical center, south west Ethiopia: prospective observational study. BMC Neurol. (2019) 19:1–11. 10.1186/s12883-019-1409-0 31390995 PMC6685251

[4] Yu JG Zhou RR Cai GJ. From hypertension to stroke: mechanisms and potential prevention strategies. CNS Neurosci. Ther. (2011) 17:577–84. 10.1111/j.1755-5949.2011.00264.x 21951373 PMC6493871

[5] Agazhe M Eshetu D Arsicha A Hamato A Petros A Dabaro D. Incidence and pattern of stroke among patients admitted to medical ward at Yirgalem General Hospital, Sidama Regional State, Southern-Ethiopia. SAGE Open Med. (2021) 9:20503121211001154. 10.1177/20503121211001154 33796298 PMC7968040

[6] Alene M Assemie MA Yismaw L Ketema DB. Magnitude of risk factors and in-hospital mortality of stroke in Ethiopia: a systematic review and meta-analysis. BMC Neurol. (2020) 20:1–10. 10.1186/s12883-020-01870-6 32814556 PMC7437163

[7] Abdu H Tadese F Seyoum G. Comparison of Ischemic and hemorrhagic stroke in the medical ward of Dessie Referral Hospital, Northeast Ethiopia: a retrospective study. Neurol Res Int. (2021). 10.1155/2021/9996958 34258063 PMC8257343

[8] Cipolla MJ Liebeskind DS Chan SL. The importance of comorbidities in ischemic stroke: impact of hypertension on the cerebral circulation. J Cereb Blood Flow Metab. (2018) 38:2129–49. 10.1177/0271678X18800589 30198826 PMC6282213

[9] Su C Liu WC Li GM Huang Y. Association between the angiotensin-converting enzyme I/D polymorphism and risk of cerebral small vessel disease: a meta-analysis based on 7186 subjects. J Stroke Cerebrovas Dis. (2021) 30:105579. 10.1016/j.jstrokecerebrovasdis.2020.105579 33412396

[10] Wang B Guo Q Peng Y Lu J Singh B Hua B. Association of AGT M235T and ACE I/D polymorphisms with the risk of ischemic stroke: meta-analysis in Han Chinese population. J Neurol Sci. (2012) 320:79–84. 10.1016/j.jns.2012.06.022 22800767

[11] Prabhakar P De T Nagaraja D Christopher R. Angiotensin-converting enzyme gene insertion/deletion polymorphism and small vessel cerebral stroke in Indian population. Int J Vasc Med. (2014). 10.1155/2014/305309 24523965 PMC3913494

[12] Ollomo B Mouélé LY Bivigou-Mboumba B Ondo BM Lendoye E Mezui J. Allele and DD Genotype of I /D Polymorphism in The ACE gene in patients with hypertension, stroke and cancer prostate in Libreville: a concern given the high frequencies of these signatures in Gabonese population. J Proteomics Genomics Res. (2018) 2:31–59.

[13] Pjević MD Bumbaširevic LB Vojvodic L Grk M Maksimović N Damnjanović T. Analysis of the association between polymorphisms within PAI-1 and ACE genes and ischemic stroke outcome after rt-PA therapy. J Pharm Pharm Sci. (2019) 22:142–9. 10.18433/jpps30339 31013014

[14] Malueka RG Dwianingsih EK Sutarni S Bawono RG Bayuangga HF Gofir A. The D allele of the angiotensin-converting enzyme (ACE) insertion/deletion (I/D) polymorphism is associated with worse functional outcome of ischaemic stroke. Int J Neurosci. (2018) 128:697–704. 10.1080/00207454.2017.1412962 29199539

[15] Mostafa MA El-Nabiel LM Fahmy NA Aref H Shreef E Abd El-Tawab F. gene in Egyptian ischemic stroke patients. J Stroke Cereb Dis. (2016) 25:2167–71. 10.1016/j.jstrokecerebrovasdis.2015.05.015 27468663

[16] Abate TW Zeleke B Genanew A Abate BW. The burden of stroke and modifiable risk factors in Ethiopia: a systemic review and meta-analysis. PLoS ONE. (2021) 16:e0259244. 10.1371/journal.pone.0259244 34723996 PMC8559958

[17] Gadekar T Dudeja P Basu I Vashisht S Mukherji S. Correlation of visceral body fat with waist–hip ratio, waist circumference and body mass index in healthy adults: a cross sectional study. Med J Armed Forces India. (2020) 76:41–6 10.1016/j.mjafi.2017.12.001 32020967 PMC6994756

[18] Unger T Borghi C Charchar F Khan NA Poulter NR Prabhakaran D. 2020 International society of hypertension global hypertension practice guidelines. Hypertension. (2020) 75:1334–5. 10.1161/HYPERTENSIONAHA.120.15026 32370572

[19] American Diabetes Association. 2. Classification and diagnosis of diabetes: standards of medical care in diabetes-−2020. Diabetes Care. (2020) 43:S14–31. 10.2337/dc20-S002 31862745

[20] Al Quran TM Bataineh ZA Al-Mistarehi AH Zein Alaabdin AM Allan H Al Qura'an A. Prevalence and pattern of dyslipidemia and its associated factors among patients with type 2 diabetes mellitus in Jordan: a cross-sectional study. Int J Gen Med. (2022) 1:7669–83. 10.2147/IJGM.S377463 36217367 PMC9547589

[21] Komala G Geetha K Kishwanth DM Nagendran R. Establishment of reference range for serum creatinine by IDMS and NIST SRM 967 traceable calibrator in government Kilpauk medical college hospital laboratory. Int J Clin Chem Lab Med. (2017) 3:23–32. 10.20431/2455-7153.0303005

[22] Birhan TA Molla MD Abdulkadir M Tesfa KH. Association of angiotensin-converting enzyme gene insertion/deletion polymorphisms with risk of hypertension among the Ethiopian population. PLoS ONE. (2022) 17:e0276021. 10.1371/journal.pone.0276021 36355860 PMC9648817

[23] Al-Hassani OM. Detection of AGT gene polymorphism in patient with hypertension in Mosul City. J Biotechnol. (2019) 4:18.

[24] Ghanie A Partan RU Indrajaya T Ali Z Saleh MI Hidayat R. The effect of angiotensin-converting enzyme gene polymorphisms in the coronary slow flow phenomenon at south sumatra, Indonesia population. Open Access Maced J Med Sci. (2020) 8:225–30. 10.3889/oamjms.2020.3802

[25] Borai IH Hassan NS Shaker OG Ashour E Badrawy ME Fawzi OM. Synergistic effect of ACE and AGT genes in coronary artery disease. Univ J Basic Appl Sci. (2018) 7:111–7. 10.1016/j.bjbas.2017.09.00319634497

[26] Mocan O Radulescu D Buzdugan E Cozma A Leucuta DC Procopciuc LM. Association between M235T-AGT and I/D-ACE polymorphisms and carotid atheromatosis in hypertensive patients: a cross-sectional study. In vivo. (2020) 34:2811–9. 10.21873/invivo.12107 32871819 PMC7652438

[27] Della-Morte D Guadagni F Palmirotta R Testa G Caso V Paciaroni M. Genetics of ischemic stroke, stroke-related risk factors, stroke precursors and treatments. Pharmacogenomics. (2012) 13:595–613. 10.2217/pgs.12.14 22462751

[28] Salem GM Gab-Allah GK. Angiotensin converting enzyme polymorphism and ischemic stroke. Neurosci J. (2020) 25:176–81. 10.17712/nsj.2020.3.20190117 32683396 PMC8015476

[29] Yuan H Wang X Xia Q Ge P Wang X Cao X. Angiotensin converting enzyme (I/D) gene polymorphism contributes to ischemic stroke risk in Caucasian individuals: a meta-analysis based on 22 case-control studies. Int J Neurosci. (2016) 126:488–98. 10.3109/00207454.2015.1036421 26000917

[30] Zhao J Qin X Li S Zeng Z. Association between the ACE I/D polymorphism and risk of ischemic stroke: an updated meta-analysis of 47,026 subjects from 105 case–control studies. J Neurol Sci. (2014) 345:37–47. 10.1016/j.jns.2014.07.023 25082780

[31] A Goyal A Saluja A Saraswathy KN Bansal P Dhamija RK. Role of ACE polymorphism in acute ischemic stroke. Neurology India. (2021) 69:1217. 10.1016/j.jns.2021.118676 34747787

[32] Al-Gazally ME Obed AF Al-Saadi AH. Effect of ACE gene polymorphism of Iraqi patients on ischemic stroke. Int J Chem Tech Res. (2016) 9:424–9.

[33] Kumar A Vivekanandhan S Srivastava A Tripathi M Padma Srivastava MV Saini N. Association between angiotensin converting enzyme gene insertion/deletion polymorphism and ischemic stroke in north Indian population: a case–control study and meta-analysis. Neurol Res. (2014) 36:786–94. 10.1179/1743132814Y.0000000335 24620983

[34] Tascilar N Dursun A Ankarali HA Mungan G Ekem S Baris S. Angiotensin-converting enzyme insertion/deletion polymorphism has no effect on the risk of atherosclerotic stroke or hypertension. J Neurol Sci. (2009) 285:137–41. 10.1016/j.jns.2009.06.016 19596363

[35] Isordia-Salas I Santiago-Germán D Cerda-Mancillas MC Hernández-Juárez J Bernabe-García M Leaños-Miranda A. Gene polymorphisms of angiotensin-converting enzyme and angiotensinogen and risk of idiopathic ischemic stroke. Gene. (2019) 688:163–70. 10.1016/j.gene.2018.11.080 30521887

[36] Tuncer N Tuglular S Kiliç G Sazci A Us Ö Kara I. Evaluation of the angiotensin-converting enzyme insertion/deletion polymorphism and the risk of Ischaemic stroke. J Clin Neurosci. (2006) 13:224–7. 10.1016/j.jocn.2005.08.005 16446094

[37] Alexander V Siregar Y Arina CA. Correlation of ACE gene polymorphism and hypertension in stroke ischemic patients. Sumatera Med J. (2020) 3:41–7. 10.32734/sumej.v3i1.3310

[38] Vijayan M Chinniah R Ravi PM Joseph AK Vellaiappan NA Krishnan JI. genotype and I allele predicts ischemic stroke among males in south India. Meta Gene. (2014) 2:661–9. 10.1016/j.mgene.2014.09.003 25606450 PMC4287818

[39] Handan AK Cengiz N Bedir A Onar MK. The relationship between angiotensin converting enzyme gene polimorphism and lacunar infarction. Harran Üniversitesi Tip Fakültesi Dergisi. (2013) 10:131–6.

[40] Rong C Xing Y Jiang X Wang J Gao B Zhao J. Angiotensin-converting enzyme gene polymorphism and middle cerebral artery stenosis in a Chinese Han population. Neural Regeneration Research. (2013) 8:1410. 25206436 10.3969/j.issn.1673-5374.2013.15.008 PMC4107760

[41] Yadav S Hasan N Marjot T Khan MS Prasad K Bentley P. Detailed analysis of gene polymorphisms associated with ischemic stroke in South Asians. PLoS ONE. (2013) 8:e57305. 10.1371/journal.pone.0057305 23505425 PMC3591429

[42] Hussain M Awan FR Gujjar A Hafeez S Islam M A. case control association study of ACE gene polymorphism (I/D) with hypertension in Punjabi population from Faisalabad, Pakistan. Clin Exp Hypertens. (2018) 40:186–91. 10.1080/10641963.2017.1356842 29058472

[43] Oscanoa TJ Cieza EC Lizaraso-Soto FA Guevara ML Fujita RM Romero-Ortuno R. Lack of association between angiotensin-converting enzyme (ACE) genotype and essential hypertension in Peruvian older people. Art Hypertension. (2020) 24:115–9. 10.5603/AH.a2020.0011 33342098

[44] Pinheiro DS Santos RS Jardim PC Silva EG Reis AA Pedrino GR. The combination of ACE I/D and ACE2 G8790A polymorphisms revels susceptibility to hypertension: a genetic association study in Brazilian patients. PLoS ONE. (2019) 14:e0221248. 10.1371/journal.pone.0221248 31430320 PMC6701835

[45] Bonfim-Silva R Guimaraes LO Santos JS Pereira JF Leal Barbosa AA Souza Rios DL. Case–control association study of polymorphisms in the angiotensinogen and angiotensin-converting enzyme genes and coronary artery disease and systemic artery hypertension in African-Brazilians and Caucasian-Brazilians. J Genet. (2016) 95:63–9. 10.1007/s12041-015-0599-5 27019433

[46] Singh M Singh AK Singh S Pandey P Chandra S Gambhir IS. Angiotensin-converting enzyme gene I/D polymorphism increases the susceptibility to hypertension and additive diseases: A study on North Indian patients. Clin Exp Hypertens. (2016) 38:305–11. 10.3109/10641963.2015.1107085 27030424

