# Corrigendum: Angiotensin-converting enzyme gene insertion/deletion polymorphism and risk of ischemic stroke complication among patients with hypertension in the Ethiopian population

**DOI:** 10.3389/fneur.2023.1223173

**Published:** 2023-05-30

**Authors:** Addisu Melake, Nega Berhane

**Affiliations:** ^1^Department of Biomedical Science, College of Health Science, Debre Tabor University, Debre Tabor, Ethiopia; ^2^Department of Medical Biotechnology, Institute of Biotechnology, University of Gondar, Gondar, Ethiopia

**Keywords:** angiotensin-converting enzyme, genotype, hypertension, ischemic stroke, polymerase chain reaction

In the published article, an author name was incorrectly written as “Nega Brhanie.” The correct spelling is “Nega Berhane.”

The authors apologize for this error and state that this does not change the scientific conclusions of the article in any way. The original article has been updated.

